# A modified protocol for rapid DNA isolation from cotton (*Gossypium* spp.)

**DOI:** 10.1016/j.mex.2019.01.010

**Published:** 2019-01-30

**Authors:** Qasim Ali, Ibrahim Bala Salisu, Ali Raza, Ahmad Ali Shahid, Abdul Qayyum Rao, Tayyab Husnain

**Affiliations:** a Centre of Excellence in Molecular Biology, University of the Punjab, Lahore-54000, Pakistan; b Department of Animal Science, Faculty of Agriculture, Federal University Dutse, P.M.B. 7156, Jigawa State, Nigeria; c Centre of Agricultural Biochemistry and Biotechnology, University of Agriculture, Faisalabad-38040, Pakistan

**Keywords:** PVP-10, polyvinylpyrrolidone, CTAB, cetyltrimethylammonium bromide, DNA extraction, CTAB, *Gossypium*, Polysaccharides, Polyphenols, Without liquid-nitrogen

## Abstract

Extraction of high-quality DNA from *Gossypium* (Cotton) species is notoriously difficult due to high contents of polysaccharides, quinones and polyphenols other secondary metabolites. Here, we describe a simple, rapid and modified procedure for high-quality DNA extraction from cotton, which is amenable for downstream analyses. In contrast to other CTAB methods, the described procedure is rapid, omits the use of liquid nitrogen, phenol, CsCl gradient ultracentrifugation, uses inexpensive and less hazardous reagents, and requires only ordinary laboratory equipment. The procedure employed the high concentration of NaCl and use of PVP-10 to rid the problems associated with polysaccharides and polyphenols, respectively. The average yield was approximately 10–15 μg of good quality DNA from 100 mg of tissue weight, which is adequate for projects, like genetic mapping or marker-assisted plant breeding. This protocol can be performed in as little as 3 h and may be adapted to high-throughput DNA isolation.

• Buffers A and B were redesigned from Paterson et al. (1993) and Porebski et al. (1997), respectively.
• Ribonuclease A was added before chloroform extraction.
• A simple, rapid and inexpensive DNA extraction method is described.

## Graphical abstract

**Figure:**
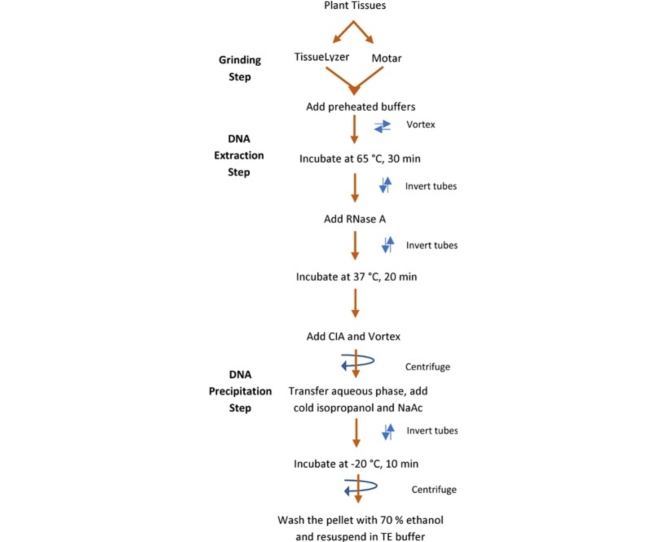

**Specifications Table**

**Table:** 

Subject area	• Biochemistry, Genetics and Molecular Biology
More specific subject area	Plant Molecular Biology
Protocol name	DNA extraction protocol
Name and reference of original method	Paterson et al. [9] and Porebski et al. [5]

## Introduction

Extraction of high-quality DNA in sufficient quantity is important for studying the molecular genetics of cotton. However, high endogenous levels of polysaccharides and polyphenols interfere with the isolation of good quality DNA, thereby rendering it unsuitable for downstream analyses [[1], [2], [3]]. During cell disruption, phenolic compounds come out of the vacuoles, become readily oxidized and irreversibly bind with nucleic acids and proteins, thus resulting in a dark DNA pellet unsuitable for most enzymatic manipulations [4]. On the other hand, the viscous nature of polysaccharides makes extracted DNA fractious to pipetting; and, it also interferes with various biological enzymes, and especially hinders the PCR reaction by inhibiting the *Thermus aquaticus* DNA polymerase (*Taq.* pol) activity [5,6].

Previously reported extraction protocols for cotton are comparatively expensive, time-consuming, require liquid nitrogen or lyophilization and ultracentrifugation in CsCl gradients [2,[7], [8], [9], [10], [11]]. Although, these methods may yield good quality DNA; however, they are not suitable for the local Pakistani varieties and high-throughput applications, such as restriction fragment length polymorphism (RFLP) and screening of transformants [9,10], that require inexpensive and reproducible DNA extractions. Another major problem for most laboratories in developing countries is the continuous procurement and storage of liquid nitrogen [12]. Over the years to overcome these problems, numerous modifications have been introduced into the original CTAB method [13] to reduce the cost and time of routine DNA isolation [14]; however, none of the modifications have been found to be universally applicable for every plant species due to their chemotypic heterogeneity. Most recent CTAB methods, including this protocol, omit the CsCl gradient ultracentrifugation, selective precipitation steps, use of liquid nitrogen and toxic phenol in favour of a simple, rapid and safe procedure.

In this regard, the described procedure was modified from Paterson et al. [9] to reduce time, cost, and resolve the problems associated with high endogenous levels of secondary metabolites, especially polysaccharides and polyphenols. This method consistently yields high-quality DNA in sufficient quantity suitable for most projects, such as cloning, mapping and marker-assisted plant breeding. An individual can routinely process 24–48 samples and isolate 10–15 μg of high-quality DNA in about 3 h. In addition, this procedure may also be adapted to a 96-well microplate format [15] for high-throughput DNA extraction.

## Materials and methods

### Plant materials

Seed and leaf tissues were harvested from the cotton variety VH-289 to test the applicability of this procedure. Approximately 3–5 cm^2^ (80–100 mg of fresh weight) of the leaf and 100 mg of the seed flour were sampled into 1.5 ml microfuge tubes and placed on ice.

### Reagents and consumables

• Cetyltrimethylammonium bromide (CTAB; Calbiochem, cat. no. 219374)
• Tris-hydrochloride (Tris–HCl; Merck-Millipore, cat. no. 108219)
• Disodium ethylenediamine (EDTA; Calbiochem, cat. no. 324503)
• Sodium dodecyl sulphate (SDS; Merck-Millipore, cat. no. 817034)
• Polyvinylpyrrolidone (PVP-10; Calbiochem, cat. no. 5295)
• 2-Mercaptoethanol (βME; Merck-Millipore, cat. no. 805740)
• Ribonuclease A (Sigma-Aldrich, cat. no. R4642)
• Ethanol absolute (Merck-Millipore, cat. no. 107017)
• Isopropanol (Merck-Millipore, cat. no. 109634)
• Sodium Acetate (NaAc; Merck-Millipore, cat. no. 106268)
• Glucose (Merck-Millipore, cat. no. 108337)
• 1.5 ml microfuge tubes and nuclease-free tips

### Solutions

• Extraction buffer A; this is modified from Paterson et al. [9]: 0.5 M glucose, 30 mM EDTA (pH 8.0), 200 mM NaCl, 200 mM Tris–HCl (pH 8.0), 0.5% (v/v) βME (add before use) and 1% (w/v) SDS
• Extraction buffer B; this is modified from Porebski et al. [5]: 2% (w/v) PVP, 2% (w/v) CTAB, 100 mM Tris−HCl (pH 8.0), 1.4 M NaCl and 20 mM EDTA (pH 8.0)
• Chloroform: isoamyl alcohol (CIA); (24:1)
• TE buffer: 1 mM EDTA (pH 8.0) and 10 mM Tris−HCl (pH 8.0)

### Equipment

• Mortar and pestle or TissueLyser (QIAGEN, cat. no. 85300) −20 °C
• Micropipettes (Eppendorf, P-200 and P-1000)
• Centrifuge (Eppendorf, cat. no. 5427 R)
• Water bath (Thermo-Scientific, cat. no. TSSWB15)
• Heat block (Marshall-Scientific, cat. no. 13259-030)

### Protocol

1 Preheat the extraction buffers A and B to 60 °C in a water bath.
2 Add 400 μl of each buffer A and B into a 1.5 ml nuclease-free microfuge tube containing 100 mg of seed flour. In case of leaf, grind 100 mg of tissue in 500 μl of buffer A using a mortar and pestle, then pour the mixture into a 1.5 ml tube. Pipette 400 μl of buffer B into the same tube.

Note: TissueLyser should be used for high-throughput extraction and to prevent cross-contamination

3 Vortex the mixture for 10 s to mix thoroughly. Incubate the tubes at 60 °C for 30 min in a water bath and invert after every 10 min to homogenize.
4 Cool down the tubes at room temperature (RT) and add RNase A (25 μg/ml). Invert the tubes for 4–5 times and incubate at 37 °C for 20 min in an incubator.
5 Add 400 μl of CIA and vortex for 5 s to form an emulsion.
6 Centrifuge at 13,000 *g* for 10 min at RT to separate the organic and aqueous phases.

Note: If the aqueous layer is not transparent then repeat the step 5.

7 Carefully transfer the aqueous (transparent) phase using a micropipette into a new tube.

Note: Wide-bore tips should be used to prevent mechanical damage to DNA.

8 Add 2/3 vol of isopropanol and 1/10 of 3.5 M NaAc (pH 5.2).
9 Close the tubes tightly, gently invert for 5–6 times and then precipitate the DNA by incubating at −20 °C for 15 min.

Note: To increase the precipitation of the DNA, the tube may be incubated for overnight.

10 Centrifuge at 13,000 *g* for 5 min to pellet the precipitated DNA.
11 Carefully remove the supernatant without disturbing the pellet and add 400 μl of 70% (v/v) chilled (−20 °C) ethanol. Dislodge the pellet by flicking with a finger.
12 Centrifuge at 13,000 *g* for 5 min and discard the supernatant by decanting.
13 Remove the ethanol residuals by drying the DNA pellet on a heat block.

Note: Do not over dry the pellet because it will make it difficult to dissolve.

14 Dissolve the DNA pellet in 40 μl of nuclease-free water.

Note: TE buffer should be used to dissolve the DNA pellet intended for long storage.

### Qualitative and quantitative analyses of the isolated DNA

A simple, rapid and comparatively cheap spectrophotometric analysis was performed to assess the purity of the extracted DNA. Ratios of UV absorption at A_260/280_ and A_260/230_ were recorded by using a Nano-Drop ND-2000 (Thermo-Scientific, USA). DNA degradation and RNA contamination were assessed through agarose gel electrophoresis.

### Restriction enzyme digestion and PCR

Approximately 10 μg of the DNA was digested with 1 unit/μg of *Hin*d III at 37 °C for 2 h, following the manufacturer’s instructions (Fermentas, USA). PCR was carried out in a thermal cycler (BIO-RAD, USA) to amplify the specific DNA sequence, in a reaction volume of 20 μl, containingPCR buffer (10 mM Tris−HCl, 50 mM KCL), 100 ng of the DNA, 1.5 mM MgCl_2_, 0.5 units of *Taq.* pol (Fermentas, USA), 0.1 mM of dNTPs (Fermentas, USA), 10 pM of each gene-specific *CP4-EPSPS* forward (5`-TATGGCTTCCGCTCAGGT-3`) and reverse (5`-AGCATCTTCTCAGTGGTCTCT-3`) primers. The amplification conditions were: initial denaturation for 5 min at 95 °C, followed by 30 cycles of 45 s denaturation at 94 °C, 45 s annealing at 52 °C and 45 s extension at 72 °C. Final extension step was at 72 °C for 10 min.

### Gel electrophoresis

2 μl of undigested and 10 μg of *Hin*d III digested DNA were subjected to electrophoresis on a 0.5 μg/ml ethidium bromide stained 1% (w/v) agarose (Thermo Scientific, USA) gel in 1X Tris-acetate-EDTA (TAE) buffer. PCR amplification was analyzed on 2% (w/v) agarose gel and documented on a GelDoc (BIO-RAD, USA).

## Results and discussion

The main steps in this method, namely cell disruption, CIA extraction and DNA precipitation, are similar to those described for other plant species [3,9,10,12,14,16,17].

The buffers used in this extraction method have been redesigned to cope with the problems associated with high levels of secondary metabolites. Specifically, the high concentration of phenol-binding reagent (PVP) and NaCl were employed to remove polyphenols and polysaccharides, respectively. Moreover, glucose was used as a reducing agent to avoid contamination and browning of the DNA pellet [18], while βME used as an antioxidant to prevent the oxidation of polyphenols. In addition, to reduce the cost and processing time of the procedure, both buffers A and B were added at same step and RNase A was added before CIA extraction.

Spectrophotometric analysis is one of the most frequently used techniques for quality assessment of a DNA. The ratio of UV absorption at A_260/280_ is 1.8 for a pure DNA, any increase in it indicates RNA contamination and conversely, the presence of protein (largely) decreases the value. The recommended absorption ratio at A_260/230_ is 2.0–2.22 for impurity free DNA [4]. The mean concentration and quality of the DNA, extracted via this method are presented in Table 1. The values of all extractions were within the accepted range, indicating a low level of contamination.

**Table 1:** Yield and purity of the genomic DNA extracted from cotton plants (± SD, n = 48).

Plant Tissues	DNA yield (μg/mg)	A_260/280_	A_260/230_	Color/Viscosity
Cotton leaf	1.5 ± 0.5	1.87 ± 0.08	2.20 ± 0.05	Clear/Non-viscous
Cotton seed	1.2 ± 0.3	1.79 ± 0.05	2.01 ± 0.03	Clear/Non-viscous

**Fig. 1:**
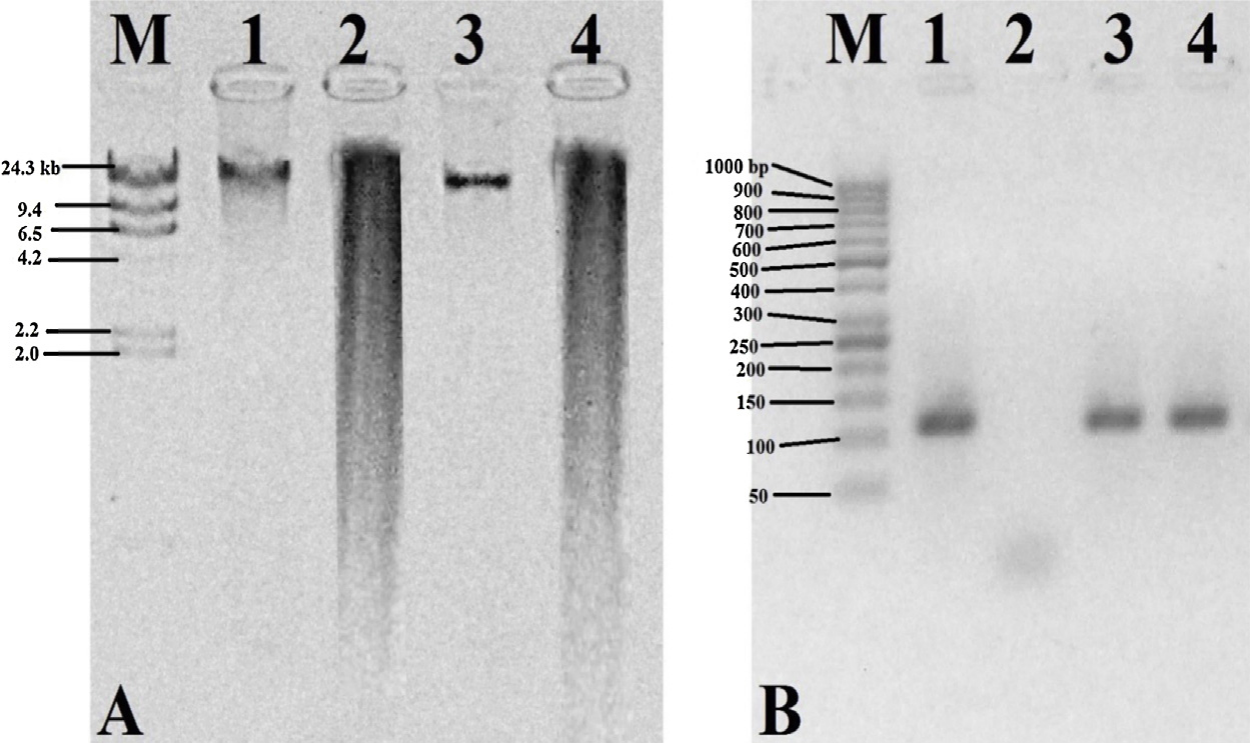
PCR, restriction and electrophoretic analyses of the isolated DNA from the seed and leaf tissues of cotton. A. 300 ng of undigested DNA and 10 μg of *Hin*d III digested DNA separated on 1% agarose gel. Lane M: λ-*Hin*d III ladder (Fermentas, USA); lane 1 and 3: undigested leaf and seed; lane 2 and 4: *Hin*d III digested leaf and seed. B. PCR amplification of the *CP4-EPSPS* gene (111 bp). Lane M: 50 bp ladder; lane 1: positive control (Recombinant plasmid DNA harboring the *CP4-EPSPS* gene sequence); lane 2: negative control (without any template DNA); lane 3 and 4: amplified sequence from leaf and seed DNA.

Being simple and efficient, agarose gel electrophoresis is quite often the method of choice to detect degradation, and impurities e.g. RNA and carbohydrates in a DNA sample [1,19]. Apart from this, DNA, that is susceptible to restriction enzymes and allows PCR amplification, is also considered to be significantly clean DNA. In this regard, the extracted DNA was further subjected to electrophoretic, PCR and restriction analyses. In result, our DNA was found to be highly susceptible to *Hin*d III restriction enzyme digestion (Fig. 1A; lane 2 and 4) and free from degradation and RNA contamination (Fig. 1A; lane 1 and 3). The PCR (111 bp) product was amplified with the gene (*CP4-EPSPS*) specific primers and separated on 2% agarose gel (Fig. 1B; lane 3 and 4). In several independent extractions or replicates, reproducible amplification and susceptibility to restriction enzyme digestion were observed.

In conclusion, we described a simple and rapid protocol that can reliably use for routine DNA isolation from cotton and is amenable for high-throughput applications, such as DNA marker-assisted selection [20,21] and cloning [22,23]. In addition, this protocol may be used for other plant species that are recalcitrant to other methods due to their high levels of polysaccharides and polyphenols.

## Conflicts of interest

None.

## Author’s contribution

QA, AQR, AAS, and TH designed the protocol. QA, IBS and AR carry out the laboratory work and optimize the method. QA wrote the manuscript and all authors revised and approved the final manuscript.
